# What Mattered Most: Personal, Work-Related, and Psychopathological Characteristics Associated with Healthcare Workers’ Impairment of Functioning during COVID-19

**DOI:** 10.3390/jcm13195821

**Published:** 2024-09-29

**Authors:** Camilla Gesi, Rita Cafaro, Matteo Cerioli, Francesco Achilli, Maria Boscacci, Giovanna Cirnigliaro, Bernardo Dell’Osso

**Affiliations:** 1 Department of Mental Health and Addiction, ASST Fatebenefratelli-Sacco, 20157 Milan, Italy; camilla.gesi@asst-fbf-sacco.it (C.G.); rita.cafaro@unimi.it (R.C.); achilli.francesco@asst-fbf-sacco.it (F.A.); maria.boscacci@asst-fbf-sacco.it (M.B.); giovanna.cirnigliaro@asst-fbf-sacco.it (G.C.); bernardo.dellosso@unimi.it (B.D.); 2 Department of Biomedical and Clinical Sciences “Luigi Sacco”, University of Milan, 20157 Milan, Italy; 3 Department of Psychiatry and Behavioural Sciences, Stanford University, Stanford, CA 94305, USA; 4 CRC “Aldo Ravelli” for Neurotechnology and Experimental Brain Therapeutics, University of Milan, 20122 Milan, Italy

**Keywords:** levels of functioning, post-traumatic stress symptoms, healthcare workers, COVID-19 pandemic

## Abstract

**Background:** The COVID-19 pandemic greatly impacted healthcare workers (HWs) around the world. Italy was the first Western country hit by the pandemic, and several studies have been published targeting the mental health burden held by Italian HWs. Notwithstanding, only a few studies focused on the impact of COVID-19 on HWs’ levels of functioning. **Methods:** An online survey was distributed to HWs in Italy through physicians’ and nurses’ associations, social networks, and researchers’ direct contacts, between 4 April and 13 May 2020. Participants provided sociodemographic, work-related, and pandemic-related data and filled out a set of psychometric questionnaires (Patient Health Questionnaire-9—PHQ-9, General Anxiety Disorder-7—GAD-7, Impact of Event Scale—Revised—IES-R, and Work and Social Adjustment Scale—WSAS). **Results:** The final sample included 1041 HWs (mean age 45.01 ± 11.62, 63.9% females). In total, 58.1% of the subjects screened positive on the GAD-7, 27.5% on the PHQ-9, and 25.9% on the IES-R. Furthermore, 67.4% showed a significant level of impairment in functioning according to the WSAS, while 35.8% reached scores of moderate or worse impairment. In the multiple linear regressions, screening positive on any of the psychometric scales and being exposed to unusual suffering significantly predicted worse scores in all WSAS domains (*p* < 0.05). Having a history of mental disorders significantly predicted worse scores in the WSAS domain of *work ability* (*p* = 0.002), while being the parent of children younger than 18 years significantly predicted worse WSAS *family functioning* scores (*p* < 0.001). **Conclusions:** Our results corroborate extant data about the impact of the COVID-19 pandemic on HWs’ mental health and shed light on its detrimental effect on functioning. Tailored interventions should be designed in order to support HWs during times of crisis.

## 1. Introduction

In February 2020, Italy became the first European hotbed of the Coronavirus Diseases (COVID-19), firstly reported between November and December 2019 in Wuhan (China). After a few weeks, the epidemic spread across the entire country, exerting an unprecedented pressure on Italian hospitals and their professional staff. Hospital overcrowding and the overwhelming workload have been abundantly described in the countries hardest hit [1,2]. Healthcare workers (HWs) worldwide have endured a great number of stressors, from the risk of being infected during work, to grueling work-shifts, to the exposure to an amount of suffering and death that was most likely unrivaled in their career until then. Several studies have shown that the COVID-19 pandemic, especially during the first wave, fostered feelings of threat, uncertainty, inadequacy, and fatigue among HWs as they had to come to terms with infection risks, changes in work tasks, worries about standards of care, lack of effective treatment or treatment guidelines, scarce or improper equipment, increased workload, and inconsistent messages from the government, the media, and hospital managers [3,4,5]. In this scenario, HWs became easily vulnerable to a broad range of psychiatric symptoms, including anxiety, depression, sleep disturbances, and post-traumatic stress symptoms, arising during or after the pandemic [6,7,8,9]. Since the COVID-19 pandemic started, several online surveys have been undertaken to evaluate the consequences on HW’s mental health, assessing diverse psychopathological dimensions [10]. It has been documented that HWs working in emergency care have been at high risk of developing post-traumatic stress disorder (PTSD), with prevalence estimates as high as 20%, especially within the Intensive Care Unit (ICU) workforce [11,12,13]. According to the most recent meta-analysis in the literature [14], which included 401 studies up to February 2022, the pooled prevalence of depression was 28.5%, while anxiety had a prevalence of 28.7%, followed by alcohol and substance use disorder (25.3%), and insomnia (24.4%). Higher odds of negative mental health outcomes were mostly associated with female gender, working on the frontline, and working in high-risk units [15].

Despite the mounting evidence documenting the large psychological response of HWs to the pandemic, studies investigating the extent of functional impairment among HWs are scant. According to Bosc, functioning implies the ability of an individual to weave effective interactions with their environment, whether it be work, family, social activities, or emotional relationships [16]. In fact, the assessment of functioning provides a comprehensive measure of a person’s ability to accomplish his or her roles, summing the contribution of several underlying causes, from personal life to work-related factors, from individual stress responses to mental resilience. Conversely, some literature points out the need to foster research and educational programs designed to enhance resilience and posttraumatic growth (PTGI) in HWs so as to be beneficial to their ability to respond to challenges in times of crisis and to experience positive psychological changes in different domains of the human experience (e.g., relating to others, personal strength, spiritual change, new opportunities, and understanding of life) [17,18]. Moreover, the assessment of functioning is essential to determine the clinical relevance of a constellation of psychological symptoms. Thus, despite the fact that the concept of functioning is complex and that the definitions may stand out as somewhat overlapping [16,19,20,21], assessing functional impairment may still provide guidance to disentangle factors that deserve to be addressed by tailored intervention from those that do not.

The aim of this paper is to assess the impairment along different dimensions of functioning among HWs during the first wave of COVID-19 in Italy. By exploring a broad set of sociodemographic, COVID-19-related, and work-related variables and the prevalence of depressive, anxiety, and post-traumatic symptoms, we tried to identify factors that were associated with higher rates of functioning impairment. A better understanding of the impact of both work-related and non-work determinants on HWs’ functioning may help us provide specific interventions in their support.

## 2. Materials and Methods

### 2.1. Study Design and Participants

In this cross-sectional study, we collected and analyzed answers from an online survey submitted to Italian healthcare workers (HWs) during the first wave of the COVID-19 pandemic. Data were collected between 4 April and 13 May in 2020, the late phase of the first stay-at-home order in Italy. An invitation to take part in the survey was addressed to HWs actively working in Italy during this period. It was sent to healthcare institutions, associations, and social network groups along with an invitation letter including a short presentation of the project. There was no exclusion in relation to rehabilitation, diagnostic, and administrative professionals. Snowball sampling was also allowed. Participants gave their informed consent to participate in the study and to have their personal data used for research purposes. Their answers were collected anonymously. The study was conducted in accordance with the principles stated in the Declaration of Helsinki. The study protocol was approved by the Department of Psychiatry of the ASST Fatebenefratelli-Sacco of Milan as the relevant institutional review board for low-risk studies (code: dsm 12-20).

### 2.2. Survey Design

The first part of the survey aimed to investigate sociodemographic (e.g., gender, age, marital, and parenthood status) and work-related variables (e.g., occupation, job seniority) and changes that occurred at home or in the workplace due to COVID-19 (e.g., changes in cohabitation status, relocation to another hospital unit). COVID-19 cases within the family or among co-workers were also assessed in this section of the survey. Previous history of mental disorders was assessed by means of a single, self-assessed question. The variable “Exposure to unusual suffering” was meant to investigate whether participants were exposed to extraordinary patients’ suffering due to COVID-19. The remaining part was meant to assess a range of psychiatric symptoms by means of a set of validated psychometric questionnaires.

### 2.3. Assessment of Anxiety Symptoms

The General Anxiety Disorder 7 (GAD-7) was used to assess the severity of anxiety symptoms. The questionnaire evaluates the presence of anxiety symptoms in the previous two weeks through seven items, scoring on a 4-point Likert scale. The GAD-7 has been extensively validated in the general population [22] and largely used to assess anxiety symptoms in healthcare providers during the COVID-19 pandemic [23]. In proceeding with previous literature, a cut-off score of 10 was used to assign a provisional diagnosis of generalized anxiety disorder [24].

### 2.4. Assessment of Depressive Symptoms

Depression was evaluated with the Patient Health Questionnaire-9 (PHQ-9), a nine-item self-report questionnaire assessing depressive symptoms in the last 15 days [25]. Each item is rated on a 4-point scale, with total scores ranging from 0 to 27. In accordance with the literature, a score of 10 or above is a reliable cut-off to assign a provisional diagnosis of major depression [26]. The PHQ-9 has been largely used as a screening tool for depression in primary care settings, as well as to assess depressive symptoms in healthcare workers during COVID-19 [27,28].

### 2.5. Assessment of Post-Traumatic Symptoms

Participants were asked to answer a gate question about possible traumatic events experienced during or because of the pandemic. Subjects providing a positive answer were invited to briefly describe the event and to fill out the Impact of Event Scale-Revised (IES-R) [29]. The scale consists of 22 items, scored on a five-point Likert scale and divided into three subscales (investigating intrusion, avoidance, and hyperarousal symptoms in the last 15 days). A score of 33 or higher on the IES-R is suggestive of a provisional diagnosis of PTSD [30]. While the IES-R is not meant to be a diagnostic tool, a total score of 33 has been indicated as having good diagnostic sensitivity (0.91) and specificity (0.82) compared to a clinical diagnosis of post-traumatic stress disorder (PTSD) [30,31]. The Italian version has also shown optimal psychometric properties and validity [32]. The IES-R has already been validated to assess PTSD during the COVID-19 pandemic [33]. In our sample, the IES-R showed good reliability (Cronbach’s alpha = 0.93). In the present study, subjects answering positive to the gate question and receiving a score of 33 or higher were given a provisional diagnosis of PTSD.

### 2.6. Assessment of Impairment of Functioning

HWs’ impairment of functioning was evaluated by means of the Work and Social Adjustment Scale (WSAS), a five-item instrument that has been extensively used and validated to assess the impact of psychiatric disorders on work and social functioning [34,35]. The first item aims to assess working ability, the second home management, the third and the fourth items social and private recreational activities, whereas the last item concerns the impairment within the family and other close relationships. Each item can score from 0 to 8, with lower scores reflecting better functioning, and a maximum total score of 40. Among subjects with depression, a WSAS score between 10 and 20 relates to a significant functional impairment, while a score above 20 indicates moderately/severe or worse impairment [34].

### 2.7. Statistical Analysis

Statistical analyses were performed using the Statistical Package for Social Science, version 27.0 (IBM Corp. Released 2020, Armonk, NY, USA). Continuous and categorical variables were reported respectively as the mean ± standard deviation (SD) and total number and percentage. Student’s *t* test was used to compare WSAS scores across a series of dichotomous characteristics of the sample and to compare WSAS scores between subjects falling above and below the cut-off scores of the GAD-7, IES-R, and PHQ-9. Five multiple linear regression models were performed with the aim of identifying significant predictors of global functioning impairment among a range of variables that showed a significant difference in the *t*-test analyses. A *p* value < 0.5 was considered statistically significant.

## 3. Results

A total of 1044 subjects completed the survey. As the survey was distributed with the assistance of healthcare institutions, associations, and social networks, the response rate could not be calculated. After data cleaning, three subjects were excluded for incomplete/inconsistent data or duplicated records. The final sample thus included 1041 HWs. The mean age was 45.01 ± 11.62 years, 665 (63.9%) were females, 455 (43.7%) were from Lombardy, and 586 (56.3%) were from other regions. The majority of the sample (77.1%) completed the survey before May 4th, the date that marks the end of the first lockdown in Italy. Among the respondents, 832 (79.9%) were physicians, 105 (10.1%) were nurses, and 104 (10.0%) were a mixed group mostly composed of midwives, rehabilitation personnel, and laboratory technicians. A total of 50 participants (4.8%) were newly employed (less than 12 months of activity), while 90 (8.6%) had a frontline role in the management of COVID-19 patients.

### 3.1. Correlates of Positive Screening to GAD-7, PHQ-9, and IES-R

Altogether, 58.1% of the subjects screened positive for generalized anxiety disorder, as assessed by scoring at least 10 on the GAD-7. Positive screening was significantly associated with participating in the survey before May 4th (*p* = 0.006), being female (*p* < 0.001), having a positive psychiatric history (*p* < 0.001), being from other regions than Lombardy (*p* = 0.035), being exposed to an unusual amount of suffering (*p* < 0.001), and having faced separation from family (*p* < 0.001). Conversely, 27.5% of the sample screened positive for major depression, as assessed by scoring at least 10 on the PHQ-9. Positive screening was significantly associated with age up to 40 years (*p* = 0.001), being female (*p* < 0.001), having a positive psychiatric history (*p* < 0.001), COVID-19 infection among close ones (*p* = 0.01), changes in work tasks (*p* < 0.001), being moved to other wards (*p* < 0.001), being moved to COVID-19 wards (*p* < 0.001), exposure to an unusual level of suffering (*p* < 0.001), and having faced separation from family (*p* < 0.001). In addition, 270 (25.9%) subjects tested positive for a provisional diagnosis of PTSD, as assessed by scoring at least 33 on the IES-R.

### 3.2. Impairment in Functioning

A total of 67.4% of subjects showed a significant level of functioning impairment according to the WSAS, while 35.8% reached scores of moderate or worse functioning impairment.

The WSAS total scores were found to be significantly higher among HWs of female gender (*p* < 0.001), younger than 40 (*p* = 0.017), responsible for a minor child (*p* = 0.005), and who were separated from their family due to fear of contagion (*p* < 0.001). Significantly higher WSAS total scores were also found among HWs who witnessed infections (*p* = 0.009) or deaths (*p* = 0.001) among close ones, those who experienced a change in tasks (*p* < 0.001), who were moved to other wards (*p* < 0.001) or to COVID-19 wards (*p* < 0.001), and who were exposed to an unusual level of suffering (*p* < 0.001). A full overview of the associations among WSAS total and subscale scores and sociodemographic, clinical, and work-related variables is shown in Table 1 and in Table 2.

A series of five linear regression models were run in order to test possible independent predictors for each WSAS domain score (see Table 3). As shown in Table 2, screening positive for any of the psychometric scales—namely, IES-R, PHQ-9, or GAD-7—significantly predicted worse scores to all WSAS domains (all *p* < 0.001). Being exposed to unusual suffering was a significant predictor of all WSAS domains scores as well (<0.05). Having a history of mental disorders significantly predicted higher scores in the WSAS assessing the impairment in work ability (*p* = 0.002), while being the parent of children younger than 18 years significantly predicted worse WSAS family functioning scores (*p* < 0.001).

## 4. Discussion

The aim of the present study was to evaluate the interplay between sociodemographic and clinical variables and global functioning in a large sample of HWs who faced the acute phase of the COVID-19 pandemic in Italy.

As expected, the presence of anxious, depressive, or post-traumatic symptoms left the greatest footprint on the levels of functioning. Specifically, HWs exceeding the cut-off scores of the PHQ-9, GAD-7, and IES-R reported significantly worse WSAS scores compared to those who did not, reporting a greater risk of impaired functioning in the multiple linear regressions as well. Other studies in the literature pointed out how, during or after disease outbreaks, HWs are at risk of mental disorders, highlighting the urge to promote interventions to help them endure the hardships and provide relief for the negative mental outcomes [3,7,9,28].

Our prevalence estimates of depression and PTSD (27.5% and 25.9%, respectively) were quite comparable to those provided by most recent meta-analyses on HWs’ mental health issues during COVID-19 [14,36], while the prevalence of generalized anxiety (58.1%) was way above the upper end of the confidence interval suggested by most of the pooled analyses from the literature (ranging from 30 to 38%). One possible explanation could be that our data started to be collected in a very precocious phase and that a big share of participants worked in a geographical area that was hardest hit by the pandemic. Therefore, it is possible that in addition to the later-developing post-traumatic, anxious, and depressive symptoms, our survey had the chance to catch the transient surge in anxiety that often represents the first psychological response to extreme stress. Most importantly, our data enriched the extant literature by showing the great impact of such conditions on the levels of functioning across different areas, including the professional one. Only a few studies, in fact, have focused on functional impairment among HWs during COVID-19. Carmassi et al. found anxiety, depression, and PTSD symptoms being among the predictors of impaired functioning, together with younger age and frontline activity, while being a medical doctor (vs. nurse/other) was shown to play a protective role for some dimensions of functioning [9]. Another study conducted in US HWs found a predictive role of PTSD and depressive symptoms, but not anxiety, on work functioning [37]. Strikingly, more than two-thirds of HWs in our sample displayed impaired functioning at a significant level, and more than one-third endorsed a level of impairment between moderate and severe. Although several unassessed factors could have contributed to the broad functional impairment found in our study, psychopathological symptoms clearly stood out as extensive and modifiable factors. As such, they should be addressed by means of screening procedures and tailored interventions, be these specific training, work–family conciliation interventions, or new tools such as telemedicine, towards which HWs can cultivate positive attitudes in terms of reliability, speed, confidentiality, and productivity [10,38,39,40].

Furthermore, some sociodemographic factors, such as female gender, younger age, and parenting a child under 18 years of age were found to be associated with greater impairment in several areas of functioning. None of these factors, however, was a significant predictor of functioning in the multiple linear regressions, except for parenting a young child. Taking care of young children has been shown to increase the risk of post-traumatic stress symptoms in a previous study based on the same dataset [28]. The present results further highlight the importance of non-work determinants in shaping the impact of massive sanitary events such as COVID-19 on healthcare personnel. Ideally, our results could additionally provide valuable hints to healthcare institutions to include work–family conciliation interventions in their policies as a better safeguard of HWs’ wellbeing and work functioning. Consistently, the results showed that being separated from or witnessing COVID-19 among close ones was associated with worse functioning.

Several work-related variables were also shown to have a detrimental impact on functioning. Most of these variables were aimed at assessing changes in daily routines due to COVID-19 (e.g., change in usual tasks; relocation to other units), while work-related variables that are stable for a long time, such as work area (frontline vs. no frontline) or role (medical doctor/nurse vs. other), did not affect the levels of functioning in a differential way. Again, while a number of variables were significantly associated with functioning in the bivariate *T* test, in the multiple linear regressions, only the exposure to unusual suffering demonstrated a significant predictive effect on all functioning domains. Taken together, these results seem to suggest that factors affecting the levels of functioning are likely rooted in the psychological burden and professional challenge posed by the unforeseen confrontation with critical patients. They also highlight the importance of adequate training for facing large-scale emergencies in the workplace. Somewhat consistently, the study by [9], conducted in Italy during COVID-19, suggested how different outcomes on functioning may depend on uneven professional training and a dearth of experience due to different roles or younger work age. Surprisingly, we did not find any correlation between frontline activity and impairment in functioning despite a body of literature supporting poorer mental outcomes and worse functioning in this group [41,42,43]. One possible explanation is that our sample included very few frontline HWs (8.9%) compared to previous studies, which may have prevented some differences from manifesting. On the other hand, our data further support the hypothesis that working on the frontline while not prepared and trained for it—instead of working on the frontline on its own—may increase the risk of worse functioning.

The findings of our study must be seen in light of some limitations. First and foremost, the data have been collected through self-reporting instruments. Second, the cross-sectional design did not allow us to infer about causality effects. Moreover, the type of recruitment may result in potential sample bias with the underrepresentation of some groups (e.g., professionals other than doctors/nurses) and overrepresentation of certain characteristics (e.g., female gender). Furthermore, although we aimed to include a large set of different groups of variables, several unassessed factors arguably played a role in HWs’ levels of functioning and might have acted as confounders in our analyses. For instance, we did not collect data about the presence of medical diseases nor about changes in voluptuary behaviors. Eventually, while some solid hints were provided by the linear regressions, further testing of our data by means of different statistical methods (e.g. mediation/moderation analysis) might help to understand the extent to which factors that we found to affect the levels of functioning exerted their detrimental effect in a direct or indirect manner. This may even better inform health institutions when designing their emergency plans and their interventions toward HWs.

## 5. Conclusions

The present paper highlights the burden of the COVID-19 pandemic on healthcare workers’ mental health, underlining a stronger association between clinical negative outcomes and functioning. Higher levels of functioning impairment were especially found among individuals with depressive, anxious, and post-traumatic stress symptoms compared to those without. Psychopathological variables were predictive factors of impairment in each functioning domain of the WSAS, controlling for a number of covariates. Additionally, exposure to an unusual amount of suffering was also a predictor of impairment in every WSAS domain. Thus, our results seem to suggest that functional impairment is mainly linked to the psychological burden and professional challenge posed by the unforeseen confrontation with critical patients. By highlighting factors that exert an actual impact on functioning, our study provides further information to design support policies and intervention strategies for HWs during large-scale events.

## Figures and Tables

**Table 1:** WSAS scores (mean +/− SD) in the total sample (n = 1041) and divided by HWs’ sociodemographic characteristics, with effect sizes calculated according to Cohen’s *d*.

		WSAS 1 Working Activities		WSAS 2 Household Chores		WSAS 3 Social Activities			WSAS 4 Private Activities		WSAS 5 Family Relationships	
	N (%)	Mean ± SD	*p*	Mean ± SD	*p*	Mean ± SD		*p*	Mean ± SD	*p*	Mean ± SD	*p*
Sex		d = 0.147		d = 0.314		d = 0.309			d = 0.346		d = 0.232	
Female	665 (63.9)	2.48 ± 1.8	**0.029**	3.4 ± 1.8	**<0.001**	3.9 ± 2.4		**<0.001**	3.9 ± 2.4	**<0.001**	3.4 ± 2.2	**<0.001**
Male	376 (36.1)	2.2 ± 1.9		2.8 ± 2.0		3.1 ± 2.4			3.1 ± 2.5		2.9 ± 2.3	
Age (yrs. old)		d = 0.154		d = 0.158		d = 0.181			d = 0.136		d = 0.068	
<40	429 (41.2)	2.6 ± 1.8	**0.020**	3.4 ± 1.9	**0.017**	3.9 ± 2.4	**0.006**		3.8 ± 2.5	**0.040**	3.3 ± 2.2	0.303
>40	612 (58.8)	2.3 ± 1.8		2.1 ± 2.0		3.4 ± 2.5			3.5 ± 2.5		3.2 ± 2.3	
Time of completion		d = 0.087		d = −0.062		d = −0.128			d = −0.067		d = −0.070	
Before May 4th	803 (77.1)	2.3 ± 1.8	0.245	3.2 ± 1.9	0.405	3.7 ± 2.4	0.087		3.7 ± 2.5	0.371	3.3 ± 2.3	0.350
After May 4th	238 (22.9)	2.5 ± 2.0		3.1 ± 2.1		3.4 ± 2.6			3.5 ± 2.6		3.1 ± 2.3	
Region		d = −0.074		d = 00.31		d = −00.83			d = −00.53		d = −00.93	
Lombardy	455 (43.7)	2.5 ± 1.9	0.260	3.2 ± 1.9	0.638	3.6 ± 2.5	0.497		3.5 ± 2.5	0.255	3.2 ± 2.3	0.594
Other	586 (56.3)	2.3 ± 1.8		3.2 ± 1.9		3.7 ± 2.4			3.7 ± 2.5		3.3 ± 2.3	
Role		d = −0.080		d = −0.075		d = −0.120			d = −00.47		d = −0.008	
Doctor/nurse	937 (90)	2.4 ± 1.8	0.446	3.2 ± 1.9	0.473	3.7 ± 2.5	0.250		3.7 ± 2.5	0.655	3.2 ± 2.3	0.939
Other *	104 (10)	2.3 ± 1.9		3.1 ± 1.9		3.3 ± 2.4			3.5 ± 2.4		3.2 ± 2.3	
Work area		d = −0.61		d = −1.88		d = −1.34			d = −0.118		d = −0.318	
Frontline	90 (8.6)	2.8 ± 2.2	**0.041**	3.4 ± 1.8	0.366	3.8 ± 2.3	0.490		3.7 ± 2.5	0.712	3.3 ± 2.3	0.792
Other °	951 (91.4)	2.3 ± 1.8		3.2 ± 2.0		3.6 ± 2.5			3.6 ± 2.5		3.2 ± 2.3	
Caregiver of minor child		d = −0.061		d = −0.188		d = −0.134			d = −0.118		d = −0.318	
Yes	652 (62.6)	2.3 ± 1.8	0.364	3.1 ± 1.9	**0.006**	3.5 ± 2.4	0.051		3.5 ± 2.5	0.86	3.0 ± 2.3	**<0.001**
No	389 (37.4)	2.5 ± 1.9		3.4 ± 2.0		3.8 ± 2.5			3.8 ± 2.5		3.7 ± 2.2	
Length of service		d = −0.019		d = −0.081		d = −0.018			d = 0.005		d = −0.245	
>1 year	991 (95.2)	2.4 ± 1.9	0.903	3.2 ± 1.9	0.593	3.6 ± 2.5	0.904		3.6 ± 2.5	0.971	3.2 ± 2.3	0.126
<1 year	50 (4.8)	2.4 ± 1.9		3.4 ± 2.0		3.7 ± 2.5			3.6 ± 2.4		3.8 ± 2.4	

* including administrative, rehabilitation, diagnostic, and technical personnel. ° including Emergency Department and Intensive Care Unit personnel.

**Table 2:** WSAS scores (mean +/− SD) in the total sample (n = 1041) and divided by HWs’ pandemic-related and clinical characteristics, with effect sizes calculated according to Cohen’s *d*.

	N (%)	WSAS 1 Working Activities		WSAS 2 Household Chores		WSAS 3 Social Activities		WSAS 4 Private Activities		WSAS 5 Family Relationships		
		Mean ± SD	*p*	Mean ± SD	*p*	Mean ± SD	p	Mean ± SD	*p*	Mean ± SD		*p*
History of mental disorders		d = −0.428		d = −0.404		d = −0.281		d = −0.319		d = −0.368		
Negative	779 (74.8)	2.2 ± 1.8	**<0.001**	3.0 ± 1.9	**<0.001**	3.4 ± 2.5	**<0.001**	3.4 ± 2.5	**<0.001**	3.0 ± 2.3	**<0.001**	
Positive	262 (25.2)	2.97 ± 1.9		3.8 ± 1.9		4.1 ± 2.4		4.2 ± 2.4		3.8 ± 2.2		
Infection among close ones		d = −0.190		d = −0.182		d = −0.183		d = −0.124		d = −10.27		
No	715 (68.7)	2.3 ± 1.8	**0.007**	3.1 ± 2.0	**0.009**	3.5 ± 2.5	**0.009**	3.5 ± 2.5	**0.076**	3.1 ± 2.3	**0.061**	
Yes	326 (31.3)	2.6 ± 1.9		3.5 ± 1.8		3.9 ± 2.5		3.8 ± 2.4		3.4 ± 2.1		
Deaths among close ones		d = −0.258		d = −0.251		d = −0.224		d = −0.156		d = −0.228		
No	762 (73.2)	2.3 ± 1.8	**<0.001**	3.1 ± 1.9	**0.001**	3.5 ± 2.4	**0.003**	3.5 ± 2.5	**0.034**	3.1 ± 2.2	**0.002**	
Yes	279 (26.8)	2.7 ± 2.0		3.6 ± 2.0		4.0 ± 2.5		3.9 ± 2.5		3.6 ± 2.3		
Changes in usual tasks		d = −2.68		d = −0.334		d = −0.323		d = −0.327		d = −0.205		
No	750 (72)	2.2 ± 1.8	**<0.001**	3.0 ± 2.0	**<0.001**	3.4 ± 2.4	**<0.001**	3.4 ± 2.4	**<0.001**	3.1 ± 2.2	**0.007**	
Yes	291 (28)	2.7 ± 2.0		3.7 ± 2.0		4.2 ± 2.5		4.2 ± 2.6		3.6 ± 2.5		
Relocation to other units		d = −0.259		d = −0.322		d = 0.327		d = 0.286		d = −0.215		
No	851 (81.7)	2.3 ± 1.8	**0.002**	3.1 ± 1.9	**<0.001**	3.5 ± 2.4	**<0.001**	3.5 ± 2.5	**0.001**	3.1 ± 2.2	**0.009**	
Yes	190 (18.3)	2.7 ± 2.1		3.7 ± 2.0		4.3 ± 2.5		4.2 ± 2.5		3.6 ± 2.5		
Relocation to COVID-19 units		d = −0.299		d = −0.359		d = −0.346		d = −0.325		d = −0.243		
No	741 (71.2%)	2.2 ± 1.7	**<0.001**	3.0 ± 1.9	**<0.001**	3.4 ± 2.4	**<0.001**	3.4 ± 2.4	**<0.001**	3.05 ± 2.2	**0.001**	
Yes	300 (28.8%)	2.8 ± 2.03		3.7 ± 1.9		4.2 ± 2.5		4.2 ± 2.5		3.6 ± 2.4		
Exposure to unusual suffering		d = −0.470		d = −0.595		d = −0.583		d = −0.513		d = −0.425		
No	614 (59%)	2.0 ± 1.6	**<0.001**	2.72 ± 1.8	**<0.001**	3.0 ± 2.2	**<0.001**	3.1 ± 2.3	**<0.001**	2.8 ± 2.2	**<0.001**	
Yes	427 (41%)	2.9 ± 2.0		3.8 ± 2.0		4.3 ± 2.5		4.3 ± 2.5		3.7 ± 2.3		
Separation from family		d = −0.305		d = −0.567		d = −0.538		d = −0.562		d = −0.473		
No	908 (87.2%)	2.3 ± 1.8	**0.002**	3.1 ± 1.9	**<0.001**	3.5 ± 2.4	**<0.001**	3.5 ± 2.4	**<0.001**	3.1 ± 2.2	**<0.001**	
Yes	133 (12.8%)	2.9 ± 2.1		4.15 ± 2.1		4.8 ± 2.6		4.8 ± 2.5		4.2 ± 2.3		
PHQ-9 screening		d = −0.967		d = −1.427		d = −1.288		d = −1.208		d = −1.095		
Negative	663 (63.7%)	2.0 ± 1.5	**<0.001**	2.5 ± 1.6	**<0.001**	2.7 ± 2.1	**<0.001**	2.8 ± 2.2	**<0.001**	2.6 ± 2.0	**<0.001**	
Positive	286 (27.5%)	3.5 ± 2.0		4.8 ± 1.7		5.5 ± 2.2		5.5 ± 2.2		4.8 ± 2.2		
GAD-7 screening		d = −0.562		d = −0.887		d = −0.748		d = −0.831		d = −0.773		
Negative	344 (33%)	1.7 ± 1.6	**<0.001**	2.2 ± 1.6	**<0.001**	2.5 ± 2.1	**<0.001**	2.4 ± 2.2	**<0.001**	2.2 ± 1.8	**<0.001**	
Positive	605 (58.1%)	2.8 ± 1.9		3.8 ± 1.9		4.3 ± 2.4		4.3 ± 2.4		3.8 ± 2.3		
**IES-R screening**		d = −0.917		d = −1.148		d = −0.958		d = −0.999		d = −0.888		
Negative	771 (74.1%)	1.94 ± 1.6	**<0.001**	2.7 ± 1.7	**<0.001**	3.0 ± 2.2	**<0.001**	3.0 ± 2.3	**<0.001**	2.7 ± 2.1	**<0.001**	
Positive	270 (25.9%)	3.50 ± 2.0		4.6 ± 1.8		5.2 ± 2.4		5.3 ± 2.3		4.6 ± 2.2		

**Table 3:** Multiple linear regression analyses for predictors of WSAS item scores, with effect sizes according to adjusted r^2^.

Factors	WSAS 1 Working Activities			WSAS 2 Household Chores			WSAS 3 Social Activities			WSAS 4 Private Activities			WSAS 5 Family Relationships		
	b (SE)	CI 95%	*p*	b (SE)	CI 95%	*p*	b (SE)	CI 95%	*p*	b (SE)	CI 95%	*p*	b (SE)	CI 95%	*p*
Adjusted r^2^	0.230			0.390			0.320			0.312			0.273		
K (constant)	1.33 (0.14)	1.05–1.62	**<0.001**	1.78 (0.13)	1.52–2.05	**<0.001**	2.11 (0.18)	1.75–2.46	**<0.001**	2.08 (0.18)	1.72–2.44	**<0.001**	1.61 (0.17)	1.27–1.95	**<0.001**
Sex	0.14 (0.12)	−0.08–0.37	0.228	−0.41 (0.11)	−0.25–0.17	0.704	−0.15 (0.15)	−0.43–0.14	0.306	−0.22 (0.15)	−0.51–0.06	0.127	0.07 (0.14)	−0.20–0.34	0.609
Age	−0.16 (0.11)	−0.38–0.06	0.149	−0.09 (0.10)	−0.29–0.12	0.406	0.14 (0.14)	−0.42–0.14	0.323	−0.02 (0.14)	−0.30–0.26	0.876	−0.02 (0.13)	−0.29–0.24	0.862
Work area	0.33 (0.20)	−0.06–0.73	0.099	0.02 (0.19)	−0.34–0.41	0.851	−0.60 (0.26)	−0.56–0.44	0.818	−0.83 (0.26)	−0.59–0.43	0.749	−0.50 (0.24)	−0.53–0.43	0.836
History of mental disorders	0.40 (0.13)	0.14–0.64	**0.002**	0.18 (0.12)	−0.05–0.42	0.121	−0.01 (0.16)	−0.32–0.31	0.977	0.08 (0.16)	−0.24–0.39	0.637	0.26 (0.15)	−0.04–0.56	0.086
Children <18 years	0.01 (0.11)	−0.21–0.23	0.932	0.19 (0.10)	−0.02–0.39	0.074	0.12 (0.14)	−0.16–0.40	0.400	0.07 (0.14)	−0.22–0.35	0.647	0.55 (0.13)	0.29–0.82	**<0.001**
COVID-19 infection among close ones	0.58 (0.13)	−0.19–0.31	0.652	0.01 (0.12)	−0.23–0.24	0.983	0.06 (0.16)	−0.26–0.37	0.731	−0.01 (0.16)	−0.33–0.31	0.955	−0.10 (0.15)	−0.40–0.21	0.541
COVID-19 death among close ones	0.21 (0.13)	−0.05–0.47	0.080	0.16 (0.12)	−0.08–0.41	0.190	0.17 (0.17)	−0.16–0.50	0.317	0.02 (0.17)	−0.31–0.35	0.900	0.25 (0.16)	−0.07–0.56	0.120
Change in tasks	0.16 (0.15)	−0.13–0.45	0.294	0.20 (0.14)	−0.07–0.47	0.148	0.22 (0.19)	−0.15–0.59	0.238	0.36 (0.19)	−0.02–0.73	0.060	0.07 (0.18)	−0.28–0.42	0.701
Relocation to other units	−0.86 (0.18)	−0.44–0.27	0.630	−0.16 (0.17)	−0.49–0.17	0.337	−0.13 (0.23)	−0.58–0.31	0.557	−0.28 (0.23)	−0.73–0.16	0.214	−0.14 (0.22)	−0.56–0.28	0.518
Relocation to COVID-19 units	0.04 (0.16)	−0.14–0.42	0.816	0.09 (0.15)	−0.20–0.38	0.534	0.10 (0.20)	−0.28–0.49	0.600	0.17 (0.20)	−0.023–0.56	0.407	0.06 (0.19)	−0.31–0.43	0.759
Exposure to unusual suffering	0.29 (0.12)	0.05–0.53	**0.020**	0.399 (0.114)	0.17–0.62	**<0.001**	0.594 (0.155)	0.290–0.898	**<0.001**	0.41 (0.16)	0.10–0.72	**0.009**	0.30 (0.15)	0.02–0.59	**0.039**
Separation from family due to COVID-19	−0.13 (0.17)	−0.46–0.20	0.436	0.14 (0.16)	−0.17–0.44	0.380	0.206 (0.211)	−0.207–0.620	0.328	0.32 (0.21)	−0.096–0.740	0.130	0.15 (0.20)	-0.24–0.54	0.458
GAD-7 screening	0.32 (0.13)	0.08–0.57	**0.009**	0.66 (0.12)	0.43–0.89	**<0.001**	0.66 (0.16)	0.35–0.97	**<0.001**	0.86 (0.16)	0.55–1.17	**<0.001**	0.79 (0.15)	0.50–1.09	**<0.001**
PHQ-9 screening	0.96 (0.14)	0.69–1.23	**<0.001**	1.48 (0.13)	1.23–1.73	**<0.001**	1.91 (0.18)	1.56–2.25	**<0.001**	1.64 (0.18)	1.29–1.98	**<0.001**	1.42 (0.17)	1.09–1.74	**<0.001**
IES-R screening	0.82 (0.14)	0.55–1.10	**<0.001**	0.78 (0.13)	0.52–1.04	**<0.001**	0.70 (0.18)	0.35–1.05	**<0.001**	0.90 (0.18)	0.54–1.25	**<0.001**	0.70 (0.17)	0.36–1.03	**<0.001**

## Data Availability

The data that support the findings of this study are available on request from the corresponding author.

## References

[1] Armocida B., Formenti B., Ussai S., Palestra F., Missoni E. (2020). The Italian health system and the COVID-19 challenge. The Lancet Public Health.

[2] Ferrara P., Albano L. (2020). COVID-19 and healthcare systems: What should we do next?. Public Health.

[3] Hennein R., Mew E.J., Lowe S.R. (2021). Socio-ecological predictors of mental health outcomes among healthcare workers during the COVID-19 pandemic in the United States. PLoS ONE.

[4] Digby R., Winton-Brown T., Finlayson F., Dobson H., Bucknall T. (2021). Hospital staff well-being during the first wave of COVID-19: Staff perspectives. Int. J. Ment. Health Nurs..

[5] Wang C., Horby P.W., Hayden F.G., Gao G.F. (2020). A novel coronavirus outbreak of global health concern. Lancet.

[6] Carmassi C., Dell’Oste V., Bui E., Foghi C., Bertelloni C.A., Atti A.R., Buselli R., Di Paolo M., Goracci A., Malacarne P. (2022). The interplay between acute post-traumatic stress, depressive and anxiety symptoms among healthcare workers functioning during the COVID-19 emergency: A multicenter study comparing regions with increasing pandemic incidence. J. Affect Disord..

[7] Muller A.E., Hafstad E.V., Himmels J.P.W., Smedslund G., Flottorp S., Stensland S.Ø., Stroobants S., Van De Velde S., Vist G.E. (2020). The mental health impact of the COVID-19 pandemic on healthcare workers, and interventions to help them: A rapid systematic review. Psychiatr. Res..

[8] Giallonardo V., Sampogna G., Del Vecchio V., Luciano M., Albert U., Carmassi C., Carrà G., Cirulli F., Dell’osso B., Nanni M.G. (2020). The impact of quarantine and physical distancing following COVID-19 on mental health: Study protocol of a multicentric italian population trial. Front. Psychiatry.

[9] Carmassi C., Pedrinelli V., Dell’Oste V., Bertelloni C.A., Cordone A., Bouanani S., Corsi M., Baidanzi S., Malacarne P., Dell’Osso L. (2021). Work and social functioning in frontline healthcare workers during the COVID-19 pandemic in Italy: Role of acute post-traumatic stress, depressive and anxiety symptoms. Riv. Psichiatr..

[10] Pappa S., Ntella V., Giannakas T., Giannakoulis V.G., Papoutsi E., Katsaounou P. (2020). Prevalence of depression, anxiety, and insomnia among healthcare workers during the COVID-19 pandemic: A systematic review and meta-analysis. Brain Behav. Immun..

[11] Hruska B., Barduhn M.S. (2021). Dynamic psychosocial risk and protective factors associated with mental health in Emergency Medical Service (EMS) personnel. J. Affect. Disord..

[12] Cameron L., McCauley M., Broek N.v.D., McCauley H. (2024). The occurrence of and factors associated with mental ill-health amongst humanitarian aid workers: A systematic review and meta-analysis. PLoS ONE.

[13] Tong J., Zhang J., Zhu N., Pei Y., Liu W., Yu W., Hu C., Sun X. (2023). Effects of COVID-19 pandemic on mental health among frontline healthcare workers: A systematic review and meta-analysis. Front. Psychol..

[14] Lee B.E.C., Ling M., Boyd L., Olsson C., Sheen J. (2023). The prevalence of probable mental health disorders among hospital healthcare workers during COVID-19: A systematic review and meta-analysis. J. Affect. Disord..

[15] Vizheh M., Qorbani M., Arzaghi S.M., Muhidin S., Javanmard Z., Esmaeili M. (2020). The mental health of healthcare workers in the COVID-19 pandemic: A systematic review. J. Diabetes Metab. Disord..

[16] Bosc M. (2000). Assessment of social functioning in depression. Compr. Psychiatry.

[17] Gesi C., Cafaro R., Achilli F., Boscacci M., Cerioli M., Cirnigliaro G., Loupakis F., Di Maio M., Dell’osso B. (2024). The relationship among posttraumatic stress disorder, posttraumatic growth, and suicidal ideation among Italian healthcare workers during the first wave of COVID-19 pandemic. CNS Spectr..

[18] Filippou A., Giannouli V. (2023). Exploring the Experiences of Integrative Psychotherapists Regarding Resilience during the COVID-19 Pandemic in Greece: An Interpretative Phenomenological Analysis. Psych.

[19] Reich K., Nemeth L.S., Mueller M., Sternke L.M., Acierno R. (2021). Psychosocial functioning in veterans with combat-related PTSD: An evolutionary concept analysis. Nurs. Forum.

[20] Flanagan J.C., Fischer M.S., Badour C.L., Ornan G., Killeen T.K., Back S.E. (2017). The Role of Relationship Adjustment in an Integrated Individual Treatment for PTSD and Substance Use Disorders Among Veterans: An Exploratory Study. J. Dual Diagn..

[21] Campbell S.B., Renshaw K.D. (2013). PTSD symptoms, disclosure, and relationship distress: Explorations of mediation and associations over time. J. Anxiety Disord..

[22] Löwe B., Decker O., Müller S., Brähler E., Schellberg D., Herzog W., Herzberg P.Y. (2008). Validation and Standardization of the Generalized Anxiety Disorder Screener (GAD-7) in the General Population. Med. Care.

[23] Rapisarda F., Vallarino M., Brousseau-Paradis C., De Benedictis L., Corbière M., Villotti P., Cavallini E., Briand C., Cailhol L., Lesage A. (2022). Workplace Factors, Burnout Signs, and Clinical Mental Health Symptoms among Mental Health Workers in Lombardy and Quebec during the First Wave of COVID-19. Int. J. Environ. Res. Public Health.

[24] Spitzer R.L., Kroenke K., Williams J.B.W., Löwe B. (2006). A Brief Measure for Assessing Generalized Anxiety Disorder The GAD-7 [Internet]. http://archinte.jamanetwork.com/.

[25] Kroenke K., Spitzer R.L., Williams J.B.W., Löwe B. (2010). The Patient Health Questionnaire Somatic, Anxiety, and Depressive Symptom Scales: A systematic review. Gen. Hosp. Psychiatry.

[26] Levis B., Benedetti A., Thombs B.D. (2019). Accuracy of Patient Health Questionnaire-9 (PHQ-9) for screening to detect major depression: Individual participant data meta-analysis. BMJ.

[27] Costantini L., Pasquarella C., Odone A., Colucci M.E., Costanza A., Serafini G., Aguglia A., Murri M.B., Brakoulias V., Amore M. (2021). Screening for depression in primary care with Patient Health Questionnaire-9 (PHQ-9): A systematic review. J. Affect. Disord..

[28] Gesi C., Cirnigliaro G., Achilli F., Cerioli M., Cafaro R., Boscacci M., Dell’osso B. (2023). The Impact of COVID-19 Pandemic First Wave on Healthcare Workers: A New Perspective from Qualifying PTSD Criterion A to Assessing Post-Traumatic Growth. J. Clin. Med..

[29] Beck J.G., Grant D.M., Read J.P., Clapp J.D., Coffey S.F., Miller L.M., Palyo S.A. (2008). The Impact of Event Scale-Revised: Psychometric properties in a sample of motor vehicle accident survivors. J. Anxiety Disord..

[30] Fattori A., Cantù F., Comotti A., Tombola V., Colombo E., Nava C., Bordini L., Riboldi L., Bonzini M., Brambilla P. (2021). Hospital workers mental health during the COVID-19 pandemic: Methods of data collection and characteristics of study sample in a university hospital in Milan (Italy). BMC Med. Res. Methodol..

[31] Creamer M., Bell R., Failla S. (2003). Psychometric properties of the Impact of Event Scale—Revised. Behav. Res. Ther..

[32] Craparo G., Faraci P., Rotondo G., Gori A. (2013). The Impact of Event Scale—Revised: Psychometric properties of the Italian version in a sample of flood victims. Neuropsychiatr. Dis. Treat..

[33] Aljaberi M.A., Lee K.-H., Alareqe N.A., Qasem M.A., Alsalahi A., Abdallah A.M., Noman S., Al-Tammemi A.B., Ibrahim M.I.M., Lin C.-Y. (2022). Rasch Modeling and Multilevel Confirmatory Factor Analysis for the Usability of the Impact of Event Scale-Revised (IES-R) during the COVID-19 Pandemic. Healthcare.

[34] Mundt J.C., Marks I.M., Shear M.K., Greist J.M. (2002). The Work and Social Adjustment Scale: A simple measure of impairment in functioning. Br. J. Psychiatry.

[35] Vázquez Morejón A.J., Vázquez-Morejón R., Conde Álvarez P. (2021). Work and Social Adjustment Scale (WSAS): Psychometric characteristics of a Spanish adaptation in a clinical population. Behav. Cogn. Psychother..

[36] Huang J., Huang Z.-T., Sun X.-C., Chen T.-T., Wu X.-T. (2024). Mental health status and related factors influencing healthcare workers during the COVID-19 pandemic: A systematic review and meta-analysis. PLoS ONE.

[37] Hendrickson R.C., Slevin R.A., Hoerster K.D., Chang B.P., Sano E., McCall C.A., Monty G.R., Thomas R.G., Raskind M.A. (2022). The Impact of the COVID-19 Pandemic on Mental Health, Occupational Functioning, and Professional Retention Among Health Care Workers and First Responders. J. Gen. Intern. Med..

[38] Sheffler J.L., Rushing N.C., Stanley I.H., Sachs-Ericsson N.J. (2016). The long-term impact of combat exposure on health, interpersonal, and economic domains of functioning. Aging Ment. Health.

[39] Pollock A., Campbell P., Cheyne J., Cowie J., Davis B., McCallum J., McGill K., Elders A., Hagen S., McClurg D. (2020). Interventions to support the resilience and mental health of frontline health and social care professionals during and after a disease outbreak, epidemic or pandemic: A mixed methods systematic review. Cochrane Database Syst. Rev..

[40] Giannouli V. (2023). eHealth before-and-during COVID-19: Does depressive symptomatology influence attitudes of cad patients, healthcare students and professionals?. Psychiatr. Danub..

[41] Liu Q., Luo D., Haase J.E., Guo Q., Wang X.Q., Liu S., Xia L., Liu Z., Yang J., Yang B.X. (2020). The experiences of health-care providers during the COVID-19 crisis in China: A qualitative study. Lancet Glob. Health.

[42] Lai J., Ma S., Wang Y., Cai Z., Hu J., Wei N., Wu J., Du H., Chen T., Li R. (2020). Factors associated with mental health outcomes among health care workers exposed to coronavirus disease 2019. JAMA Netw. Open.

[43] Sanghera J., Pattani N., Hashmi Y., Varley K.F., Cheruvu M.S., Bradley A., Burke J.R. (2020). The impact of SARS-CoV-2 on the mental health of healthcare workers in a hospital setting—A Systematic Review. J. Occup. Health.

